# Fatal Results from the Inhalation of Chloroform

**Published:** 1851-04

**Authors:** 


### ARTICLE XIII.

### *Fatal Results from the Inhalation of Chloroform.*

As anæsthetic agents are now being much employed in the practice of dental surgery, we subjoin an interesting and instructive account relative to a post mortem examination in a fatal case in England, from the inhalation of chloroform, which will at once convince the most sceptical that great care, judgment and caution, combined with great experience, are necessary, before these agents can or ought to be universally employed.

Dr. Glover, in a communication to the *London Lancet*, says: Dr. Simpson endeavors to show that death was not caused by chloroform, but by ordinary suffocation, or "drowning," the drowning being produced by one or two tea-spoonfuls of brandy and water: (apparently, Clarence might have dispensed with a butt of malmsey for his occasion.)

The principal objections of Professor Simpson to our conclusion—viz. that the chief cause of death was the congestion of the lungs, occasioned by the inhalation of chloroform—appear to be, 1st. That the appearances observed were those of asphyxia; 2dly. That they were not such as are produced by chloroform, because in animals poisoned by chloroform-vapor, the blood is coagulated in the right side of the heart—in asphyxia, fluid; and that reddening of the epiglottis is a sign of asphyxia, not of chloroform poisoning; and that the brain is not congested in chloroform poisoning, but is so in asphyxia.

I shall relate the principal facts observed in some recent experiments, without stating everything observed.

In the presence of my friend, Mr. Sheill, I poisoned a terrier dog with chloroform-vapor, and drowned another. I also performed a similar experiment upon two rabbits.

The chloroformed dog inhaled the chloroform from a kind of nozzle, open at the end, where there was a sponge impregnated with chloroform. Death could not take place from mere want of air. He appeared pretty nearly dead in two minutes and a half, but all signs of life were extinct within seven minutes. The symptoms were, at first, considerable struggling and a little wheezing, violent and irregular motion of the heart; then, forcible action of the abdominal muscles, stertorous breathing, loss of power over the limbs, and discharge of urine and feces; heart's action very feeble, pupils much dilated. All this applies to the first two minutes and a half. During the remaining period, a slight movement of the respiratory muscles was all that could be observed.

The drowned dog struggled under water for about two minutes.

On inspection, after the interval mentioned, both dogs were stiff, and limbs extended, pupils in the drowned more dilated.

In the chloroformed, the blood was almost quite fluid in the large veins; heart distended with dark blood, mostly coagulated in both its cavities; the left lung, (the animal had been laid on its left side,) was especially congested, it was enormously so; the other lung was also very much congested, but the left was in some places like liver. Both lungs were mottled and full of blood; some bloody froth was found in the bronchi; the trachea and larynx, also the epiglottis, redder than natural.

In the drowned, the blood was more coagulated in the great veins, but less so in the heart, than in the chloroformed; but the blood was by no means quite fluid in the heart. It was rather of a treacly consistence, very dark, and there were numerous coagula—very little blood on the left side. The lungs were congested in a very different manner from what appeared in the chloroformed dog. They were certainly much congested, but the congestion was more livid, not so florid; in patches, not uniform; and there was much froth, and apparently water, in the lungs. The larynx and epiglottis were not so vascular. Soot arising from the rain water in the tub in which the drowning was effected, could be traced distinctly in the larynx and into the lungs.

The abdominal organs were rather congested in both cases; the bladder, empty in the chloroformed, contained a little water in the drowned; the membranes of the brain, and organ itself, more vascular in the chloroformed.

In experiments by Orfila, on corpses placed in water in which animal charcoal was mixed, the charcoal was found in the air passages and lungs.—*Traitê de Medicine Legali*, t. ii, 3d ed., p. 385.

That the blood is not necessarily fluid in the heart in asphyxia produced by drowning, is shown by the observations of Lafosse and Avisard, stated by Orfila. (Ibid. p. 391.) "Lafosse found the blood polypous and concrete in some drowned persons. We have found, in reality, once only, some fibrinous clots in the blood of a drowned person, and M. Avisard says that he has seen it coagulated or semi-coagulated in the right auricles and ventricles of two individuals drowned while living." In a rabbit which I drowned, the blood, twenty-four hours after death, was found in solid coagulated masses, both on the right and left sides of the heart, (auricles and ventricles,) and in large veins. At the time when I drowned this rabbit,

I also chloroformed another, in the presence of Mr. Sheill, (who also saw both rabbits opened.) It was killed in a few seconds, apparently as speedily as by prussic acid; the principal differences were the following: the blood was more solid in the drowned; the lungs presented appearances similar to those observed in the dog's; the larynx, trachea, brain and its membranes, much more vascular in the chloroformed.

In another very strong dog, which I killed with the vapor of chloroform in about fifteen minutes, on immediate inspection, the same enormous congestion of the lungs was observed; congestion of the brain, much serum in the ventricles, very considerable reddening of the larynx, trachea and epiglottis; irritability of the voluntary muscles diminished, peristaltic action went on. Heart enormously distended with dark fluid blood, principally on the right side; its irritability destroyed.

No inference can be drawn from the state of the abdominal organs, as to the cause of death by chloroform or ordinary asphyxia.

In Dr. Jamieson's case, at Aberdeen, the post-mortem appearances were essentially similar to those observed in the case of Hannah Greener; also in the recent case of poisoning by ether in France. What they were in the fatal case at Birkenhead does not yet appear, but death seems to have been produced by pulmonary apoplexy. According to Dr. Simpson's explanation of the Aberdeen case, as given in the *Scotsman*, the person contrived to fall forward on a wet towel, (wet, too, by a volatile fluid,) so cleverly, as to seal hermetically both nose and mouth!

I agree with Dr. Jamieson, that the subject of his paper died from asphyxia produced by chloroform. No doubt the appearances which occur in chloroform poisoning are very similar to those produced by ordinary asphyxia, (speaking generally;) but there remains the difference in the character of the pulmonary congestion. But I believe that chloroform may kill by syncope, by its effect in stopping the heart's action, when we may have no congestion of the lung. With regard to Professor Simpson's assertion, that the blood is *always* coagulated in the heart in death from chloroform, fluid in asphyxia, we see that it is neither uniformly fluid nor uniformly solid in either case. It was fluid in Dr. Jamieson's case, and in the recent fatal ether case in France.

Professor Simpson's assertion, that reddening of the larynx and epiglottis, and congestion of the brain, do not occur in chloroform poisoning, is shown to be erroneous.

Two other arguments require to be stated against Professor Simpson's assertions.

He makes out, I think, that Hannah Greener was hardly properly under the influence of chloroform. But if this were the case, how came the reflex phenomena of both larynx and pharynx to be destroyed? which would necessarily be the case, according to the Professor's view of the cause of death. This argument has been touched on by a recent correspondent of the Lancet. Or how could, as Dr. Meggison observes, even choking produce such enormous congestion of the lungs, when the heart was not acting?

Sir John Fife and I were of opinion that the congestion of the lung was the cause of death, because it was so enormous as to cast every other post-mortem appearance into the shade. Of the power of chloroform to produce this congestion, I warned the profession in a letter, published in the *Medical Gazette*, of December 3d, when all was *couleur de rose* concerning chloroform; and the experiments of Mr. Wakley, &c., have abundantly established the reality and formidable nature of this power. Nevertheless, I give no opinion as to the use of chloroform. I believe it to be both more powerful and more dangerous than ether. In a recent case here of a formidable character, I advised a friend to use it. We could hardly expect so great a boon as the removal of pain in surgical operations, without a *per contra*.

